# The Presence of Neutrophil Extracellular Traps (NETs) in Brain Tumor Vessels Is Linked to Platelet Aggregates and Podoplanin in the Tumor Microenvironment

**DOI:** 10.3390/cancers17193141

**Published:** 2025-09-27

**Authors:** Pegah Mir Seyed Nazari, Öykü Özer, Thomas Roetzer-Pejrimovsky, Maximilian J. Mair, Julia Riedl, Christine Brostjan, Anna Sophie Berghoff, Matthias Preusser, Johannes A. Hainfellner, Christine Marosi, Ingrid Pabinger, Cihan Ay

**Affiliations:** 1 Division of Haematology and Haemostaseology, Department of Medicine I, Medical University of Vienna, 1090 Vienna, Austria; pegah.mirseyednazari@gmail.com (P.M.S.N.); oeykue.oezer@meduniwien.ac.at (Ö.Ö.); julia.riedl@meduniwien.ac.at (J.R.); ingrid.pabinger@meduniwien.ac.at (I.P.); 2 Department of Biomedical Imaging and Image-Guided Therapy, Medical University of Vienna, 1090 Vienna, Austria; 3 Division of Neuropathology and Neurochemistry, Department of Neurology, Medical University of Vienna, 1090 Vienna, Austria; thomas.roetzer@meduniwien.ac.at (T.R.-P.); johannes.hainfellner@meduniwien.ac.at (J.A.H.); 4 Division of Oncology, Department of Medicine I, Medical University of Vienna, 1090 Vienna, Austria; maximilian.mair@meduniwien.ac.at (M.J.M.); anna.berghoff@meduniwien.ac.at (A.S.B.); matthias.preusser@meduniwien.ac.at (M.P.); christine.marosi@meduniwien.ac.at (C.M.); 5 Division of Vascular Surgery, Department of General Surgery, Medical University of Vienna, 1090 Vienna, Austria; christine.brostjan@meduniwien.ac.at

**Keywords:** podoplanin, neutrophil extracellular traps (NETs), cancer-associated thrombosis, glioma, platelet activation

## Abstract

**Background**: Multiple mechanisms might lead to cancer-related hypercoagulability. In brain tumors, podoplanin, via its ability to activate platelets, seems to play a crucial role in developing venous thromboembolism (VTE). Different stimuli (including activated platelets) can trigger the release of prothrombotic neutrophil extracellular traps (NETs) by neutrophils. It remains to be elucidated whether podoplanin-induced platelet aggregates might also impact NET formation and subsequent hypercoagulability and thrombosis. **Methods**: Patients with glioma were enrolled in this prospective observational cohort study. The primary endpoint was VTE. Immunohistochemical staining of NETs (via citrullinated histone H3 [H3Cit]) and neutrophils (via myeloperoxidase [MPO]) was conducted in glioma specimens and correlated with intravascular platelet clusters (via CD61) and podoplanin. **Results**: In total, 154 patients were included. H3Cit+ tumor vessels were found in 45/154 cases. H3Cit were significantly associated with increased intravascular platelet clusters (CD61− vs. CD61+ vs. CD61++ vs. CD61+++: 3.7% (1/27) vs. 18.6% (11/59) vs. 39.4% (13/33) vs. 57.1% (20/35), *p* < 0.001) and podoplanin expression (PDPN− vs. PDPN+: 14.3% (7/49) vs. 36.2% (38/105), *p* = 0.007) in the tumor tissue. Furthermore, H3Cit+ tumor vessels were significantly associated with tumor-infiltrating MPO+ neutrophils (H3Cit− vs. H3Cit+, median [Q1-Q3]: 6.0 [3.3–12.3] vs. 12.5 [5.9–22.0] cells/mm^2^, *p* < 0.001) and with D-dimer levels (H3Cit− vs. H3Cit+: 0.53 [0.32–1.10] vs. 0.84 [0.46–2.75] µg/mL, *p* = 0.034). The VTE risk was not linked to H3Cit+ tumor vessels (*p* = 0.613, log-rank). **Conclusions**: H3Cit in tumor vessels was not associated with VTE. However, H3Cit was linked to a local procoagulant phenotype in glioma, thereby potentially contributing to a systemic hypercoagulable state and thrombus formation.

## Simple Summary

Several mechanisms can induce cancer-associated thrombosis. In brain tumors, podoplanin upregulation on cancer cells appears to play a pivotal role in the development of venous thromboembolism (VTE). Of particular note, podoplanin is able to activate platelets via the C-type lectin-like receptor 2 (CLEC-2). Generally, inflammation is also involved in clot formation. For example, so-called neutrophil extracellular traps (NETs) can be released by neutrophils upon multiple triggers (e.g., activated platelets) and then lead to venous thrombosis. In this study, prothrombotic NET components in brain tumor vessels were linked to local procoagulant characteristics such as intravascular platelet clusters and podoplanin expression. These results highlight a possible relationship between podoplanin-induced platelet activation and NET formation, which might enhance hypercoagulability and thrombus development.

## 1. Introduction

Venous thromboembolism (VTE) is a common life-threatening vascular complication amongst cancer patients [1,2]. Patients with malignant primary brain tumors have a particularly high VTE risk [3]. The reported incidence of VTE in patients with brain tumors is up to 20–30% [4,5]. In the first year after brain cancer diagnosis, approximately 60% of the VTE cases were described as deep vein thrombosis (DVT), mostly in the lower extremity, while nearly 40% of the VTE cases were classified as pulmonary embolism (PE) [6]. So far, clinical factors, tumor-specific characteristics (e.g., isocitrate dehydrogenase 1 [IDH1] wildtype status), and systemic blood parameters (e.g., leukocytes, decreased platelet counts, soluble P-selectin, D-dimer) have been identified as VTE risk factors in these patients [7,8,9,10,11,12]. Also, tumor-expressed podoplanin was linked to an increased risk of VTE in patients with primary brain tumors [7]. Podoplanin is a sialomycin-like glycoprotein that leads to activation of platelets via the C-type lectin-like receptor 2 (CLEC-2), and its upregulation in brain tumors has been linked to increased intravascular platelet clusters in the tumor microenvironment [7,13,14]. In vitro experiments revealed that platelet activation induced by podoplanin-expressing glioblastoma cells was inhibited upon addition of a podoplanin-specific antibody, proposing a crucial role of podoplanin in the development of VTE in brain tumor patients [7]. In line, a murine glioma model confirmed that podoplanin deletion results in reduced intratumoral platelet aggregates [15]. Additionally, several in vivo studies suggested a mechanistic role for podoplanin and CLEC-2 in thrombus formation [16,17,18,19,20,21].

Neutrophils are the most abundant immune cells in the blood circulation and are important players of the innate immune system [22]. Also, cancer-promoting and cancer-suppressing functions of neutrophils have been described [23,24]. In glioma, tumor-infiltrating neutrophils were altogether associated with a higher tumor grade and worse survival [25,26]. Different stimuli (e.g., bacterial pathogens, cytokines, activated platelets, cancer cells) can lead to NETosis of neutrophils, which causes the release of so-called neutrophil extracellular traps (NETs) [22,27,28,29,30,31]. The involvement of NETs has been demonstrated in several pathophysiological processes, including fighting pathogens, tumor progression, and (cancer-associated) thrombosis [27,32,33,34,35,36,37,38,39,40,41,42]. NETs consist of several components, such as antimicrobial enzymes (e.g., myeloperoxidase [MPO], neutrophil elastase [NE]) and decondensed DNA filaments that are associated with histones (e.g., citrullinated histone H3 [H3Cit]) [22,27]. Several NET components function as procoagulants; for instance, DNA and histones are able to induce thrombin generation, and the NE enzyme is able to increase tissue factor activity by inactivation of the tissue factor pathway inhibitor [22,43,44,45,46,47,48]. After their release, NETs can build a mesh-like structure capturing platelets, leukocytes, and erythrocytes, which can lead to (micro-) thrombosis and occlusion of the vasculature system, which, amongst others, enable the limitation of the spread of invading microbes throughout the body [41,48,49,50]. Peptidylarginine deiminase 4 (PADI4) is an important enzyme with a key role in histone citrullination (e.g., H3Cit) and NET formation, and PADI4 deficiency in mice resulted in reduced thrombus formation upon inferior vena cava stenosis [51].

Interestingly, NETs can induce platelet activation and vice versa [22,29,52]. In particular, upon platelet activation, P-selectin becomes exposed on the platelet cell surface and is then able to bind to P-selectin glycoprotein ligand (PSGL)-1 on neutrophils. Eventually, this interaction can trigger the formation of NETs in neutrophils [29,53,54]. So far, it remains to be elucidated whether podoplanin-induced platelet activation might trigger the release of NETs as well, which in turn might contribute to a hypercoagulable state and subsequently thrombus formation in glioma patients. In the current study, we aim to reveal a potential link between podoplanin, platelet aggregation, and NETosis in patients with glioma. Thus, we investigated the presence of NETs in brain tumor vessels and their association with podoplanin expression and intravascular platelet clusters in the tumor microenvironment. Furthermore, we analyzed the correlation between NETs in brain tumor vessels and the risk of developing VTE.

## 2. Materials and Methods

### 2.1. Study Cohort

This study was conducted in the frame of the Vienna Cancer and Thrombosis Study (CATS), a prospective and observational single-center cohort study at the Medical University of Vienna (MUV) in Austria, which was approved by the ethics committee (number 126/2003) in accordance with the Declaration of Helsinki. The detailed study design has been published in prior publications [7,55]. The primary endpoint of the study was VTE development. VTE events were established in cases of objectively confirmed VTE. Overall, the included patients were not routinely screened. However, in the presence of VTE symptoms, objective diagnostic procedures (e.g., duplex sonography or venography for DVT and CT or ventilation/perfusion lung scan for PE) were performed in order to confirm the VTE diagnosis. In addition, accidentally detected VTE without any symptoms (such as PE) in routinely performed CT scans were included as well. Autopsy protocols were also reviewed after a patient died. In case of fatal PE, autopsy reports were used to confirm the diagnosis. The current cohort consisted of patients (≥18 years) with newly diagnosed glioma or regrowth after previous brain tumor surgery. Only patients (recruited from 2003 until 2014) with available brain tumor tissue blocks were included in the current analyses. Several exclusion criteria were defined, including chemotherapy within the last 3 months, radiotherapy or surgery within the prior 2 weeks, overt bacterial or viral infection within the last 2 weeks, as well as thromboembolic events within the last three months, and continuous prophylactic or therapeutic anticoagulation at the time of study inclusion. At the time of recruitment, a venous blood sample was collected via sterile venipuncture using Vacutainer K3-EDTA tubes (Vacuette, Greiner BioOne, Kremsmuenster, Austria). Blood cell counts were obtained from a Sysmex XE-5000 hematology analyzer (Sysmex Corporation, Kobe, Hyogo, Japan). Data on plasma levels of D-dimer and soluble P-selectin are available from our previous studies [7,55,56].

Patients included in this study were recruited before the newest versions of the WHO classification system of central nervous system (CNS) tumors [57,58]. Therefore, all gliomas were classified according to the WHO classification of 2007 [59].

### 2.2. Immunohistochemistry

Formalin-fixed and paraffin-embedded (FFPE) tissue blocks of glioma were cut into serial 3–5 µm slices and then analyzed with a Ventana Benchmark Ultra immunostaining system. The presence of NETs in vessels within the brain tumor was investigated with the polyclonal rabbit anti-histone H3 (citrulline R2 + R8 + R17) antibody; ab5103, Abcam, Cambridge, MA, USA). After immunohistochemical staining, all specimens were reviewed on a multi-headed microscope. Tumor specimens were classified as H3Cit-positive when H3Cit staining was detected in at least small and/or isolated brain tumor vessels. To assess tumor-infiltrating neutrophils, we applied a rabbit polyclonal antibody A0398 (Dako, Agilent Technologies, Inc., Santa Clara, CA, USA, 1:100) against MPO. After immunohistochemical staining, tumor slides were digitized with a NanoZoomer slide scanner (Hamamatsu Photonics, Hamamatsu, Japan). Computer-based quantification of tumor-infiltrating MPO+ neutrophils was conducted using Definiens tissue Studio V.4.4.3 (Definiens AG, Munich, Germany). Prior to measurement, necrotic areas were excluded. Density of tumor-infiltrating MPO+ neutrophils is given as cells/mm^2^ tumor tissue.

Immunohistochemical staining against IDH1 R132H mutation, podoplanin and the platelet surface protein CD61 were performed in our previous studies [7,8]. In brief, the IDH1 R132H mutation was analyzed using a monoclonal anti-IDH1 R132H antibody (Dianova, Hamburg, Germany). Podoplanin expression in tumor specimen was assessed with the monoclonal mouse anti-PDPN antibody D2-40 (Cell Marque, Rocklin, CA, USA). Tumor specimens were classified as podoplanin positive, when at least 50% of tumor cells expressed podoplanin and/or when tumor cells showed at least mild staining intensity. Intravascular platelet clusters were investigated via staining with a monoclonal mouse anti-CD61 antibody (NCL-CD61-308, Leica Biosystems, Newcastle, UK) and then semi-quantitatively classified into four levels: negative (−); isolated, small, CD61+ tumor vessels (+); multiple CD61+ tumor vessels (++); very large and/or plenty of CD61+ tumor vessels (+++).

### 2.3. Statistical Analysis

Statistical analyses were conducted with IBM SPSS Statistics (Version 30.0, IBM, Armonk, NY, USA). Categorical variables were summarized as absolute frequencies (%) and continuous variables as medians (25th–75th percentile). Differences between categorial variables were assessed with χ^2^-tests and Fisher’s exact tests, and continuous variables with the non-parametric Mann–Whitney U-tests or Kruskal–Wallis tests. The observation period was defined from study entry until VTE occurrence, death, or censoring alive within two years. The median follow-up was estimated using the reverse Kaplan–Meier method according to Schemper et al. [60]. Hazards of VTE were analyzed with uni- and multivariable Cox regression models. Furthermore, VTE probabilities were calculated with 1-Kaplan–Meier estimators. Between groups, VTE incidences were compared with log-rank tests. As a cut-off for statistical significance, *p*-values lower than 0.05 were considered significant.

## 3. Results

### 3.1. Baseline Characteristics of the Study Population

In total, 154 patients with glioma were included in the current study (Table 1). Median age of the study population was 55 years [Q1–Q3: 44–66], and 61.7% (95/154) of the patients were male. All gliomas were classified according to the 2007 WHO classification of CNS tumors [59]. Overall, 77.3% (119/154) of the patients were diagnosed with a glioblastoma (WHO grade IV), 18.2% (28/154) of the patients had an anaplastic glioma (WHO grade III), and 4.5% (7/154) of the patients were diagnosed with a diffuse glioma (WHO grade II). IDH1 mutation was present in 21.4% (33/154) of the patients. Podoplanin expression was detected in 68.2% (105/154) of the tumor specimens, while 31.8% (49/154) tumor specimens stained negative for podoplanin. Overall, 17.5% (27/154) of the tumor specimen had no intravascular CD61+ platelet clusters (−), whereas 38.3% (59/154) of the tumor specimen showed isolated, small, CD61+ tumor vessels (+), 21.4% (33/154) of the tumor specimen showed multiple CD61+ tumor vessels (++) and 22.7% (35/154) had very large and/or plenty of CD61+ tumor vessels (+++) within the tumor. Representative microscopic images of IDH1 mutation, podoplanin expression, and intravascular CD61+ platelet clusters are shown in Supplementary Figure S1.

### 3.2. Association of IDH1 Mutation with NETs in Tumor Vessels and Tumor-Infiltrating Neutrophils

**Figure 1:**
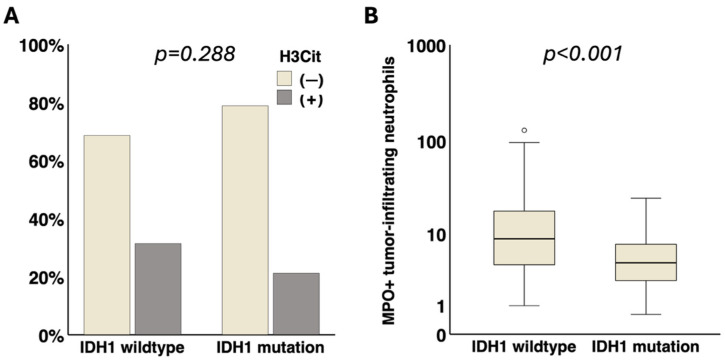
Association of IDH1 status with presence of NETs in brain tumor vessels and tumor-infiltrating neutrophils. (**A**) No significant association of H3Cit in brain tumor vessels with IDH1 status (*p* = 0.288) was detected. (**B**) Tumor-infiltrating MPO+ neutrophils were significantly linked to IDH1 wildtype status (*p* < 0.001). IDH1, isocitrate dehydrogenase 1. NETs, neutrophil extracellular traps. H3Cit, citrullinated histone H3. MPO, myeloperoxidase. ° = outlier.

Via immunohistochemistry, the presence of H3Cit in brain tumor vessels was detected in 29.2% (45/154) of the tumor specimens. Representative microscopic images of H3Cit+ brain tumor vessels are shown in Supplementary Figure S2A–B. H3Cit+ brain tumor vessels were not significantly associated with IDH1 mutation (IDH1 mutation vs. IDH1 wildtype: 21.2% (7/33) vs. 31.4% (38/121), *p* = 0.288) (Figure 1).

The median density of MPO+ neutrophils in the brain tumor tissue was 7.5 cells/mm^2^ (Q1–Q3: 3.7–15.0). Representative microscopic images of tumor-infiltrating MPO+ neutrophils are shown in Supplementary Figure S2C. Tumor-infiltrating MPO+ neutrophils were significantly associated with an IDH1 wildtype status (IDH1 mutation vs. IDH1 wildtype: median [Q1–Q3]: 4.5 [2.3–7.8] vs. 8.8 [4.2–18.0] cells/mm^2^, *p* < 0.001) (Figure 1).

### 3.3. Association of NETs in Tumor Vessels and Tumor-Infiltrating Neutrophils with Podoplanin and Intravascular Platelet Clusters in the Tumor Microenvironment and (VTE-Related) Blood Parameters

**Figure 2:**
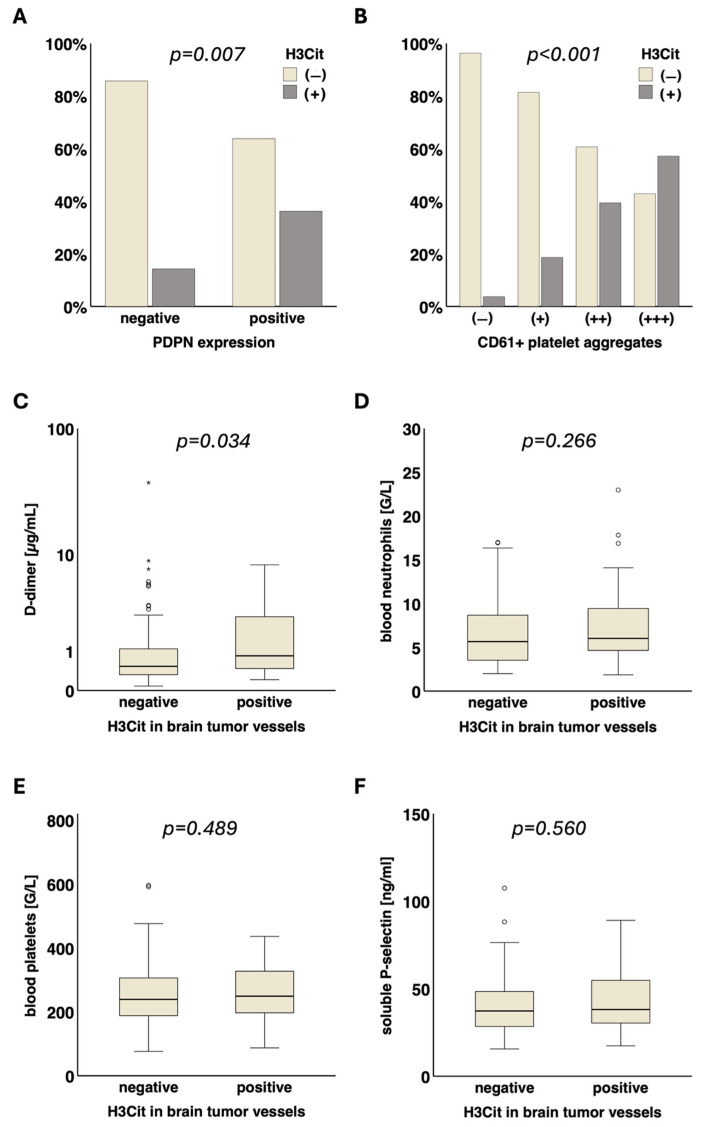
Association of NETs in brain tumor vessels with local and systemic prothrombotic characteristics. The presence of H3Cit in tumor vessels was significantly associated with (**A**) podoplanin (*p* = 0.007) and (**B**) intravascular CD61+ platelet clusters (*p* < 0.001) in the tumor microenvironment. Furthermore, H3Cit in tumor vessels was significantly linked to elevated (**C**) D-dimer levels (µg/mL, *p* = 0.034) in the blood. No significant association of H3Cit in tumor vessels with (**D**) neutrophil blood counts (G/L, *p* = 0.266) and other VTE-related blood parameters was detected: (**E**) platelets (G/L, *p* = 0.489), (**F**) soluble P-selectin (ng/mL, *p* = 0.560). NETs, neutrophil extracellular traps. H3Cit, citrullinated histone H3. MPO, myeloperoxidase. PDPN, podoplanin. VTE, venous thromboembolism. ° = outlier. * = far out outlier.

The presence of H3Cit in brain tumor vessels was significantly associated with intratumoral podoplanin expression (PDPN− vs. PDPN+: 14.3% (7/49) vs. 36.2% (38/105), *p* = 0.007) as well as with intravascular platelet clusters (CD61− vs. CD61+ vs. CD61++ vs. CD61+++: 3.7% (1/27) vs. 18.6% (11/59) vs. 39.4% (13/33) vs. 57.1% (20/35), *p* < 0.001). Moreover, H3Cit+ brain tumor vessels were significantly associated with D-dimer levels in the blood (H3Cit− vs. H3Cit+, median [Q1–Q3]: 0.53 [0.32–1.10] vs. 0.84 [0.46–2.75] µg/mL, *p* = 0.034). No significant association of H3Cit+ tumor vessels with blood platelets (H3Cit− vs. H3Cit+, median [Q1–Q3]: 239 [187–309] vs. 249 [197–327] G/L, *p* = 0.489), soluble P-selectin (H3Cit− vs. H3Cit+, median [Q1–Q3]: 37.3 [28.4–48.6] vs. 38.2 [30.2–55.3] ng/mL, *p* = 0.560), blood neutrophils (H3Cit− vs. H3Cit+, median [Q1–Q3]: 5.6 [3.5–8.7] vs. 6.0 [4.6–9.5] G/L, *p* = 0.266) and leukocytes (H3Cit− vs. H3Cit+, median [Q1–Q3]: 7.7 [5.6–10.8] vs. 8.1 [6.5–11.7] G/L, *p* = 0.276) was found (Figure 2).

The combination of H3Cit and podoplanin in glioma and their association with different laboratory parameters were assessed in subgroup analysis (Supplementary Table S1). D-dimer levels were highest in patients with both H3Cit+ tumor vessels and podoplanin expression (H3Cit−/PDPN− vs. H3Cit−/PDPN+ vs. H3Cit+/PDPN− vs. H3Cit+/PDPN+, median [Q1–Q3]: 0.37 [0.22–0.61] vs. 0.84 [0.43–1.88] vs. 0.34 [0.21–3.15] vs. 0.94 [0.51–2.77] µg/mL, *p* < 0.001). The platelet count was lower in patient subgroups with podoplanin-positive gliomas (with or without H3Cit in tumor vessels) compared to those having podoplanin-negative gliomas (H3Cit−/PDPN− vs. H3Cit−/PDPN+ vs. H3Cit+/PDPN− vs. H3Cit+/PDPN+, median [Q1–Q3]: 283 [240–362] vs. 213 [173–265] vs. 335 [318–350] vs. 237 [186–308] G/L, *p* < 0.001).

**Figure 3:**
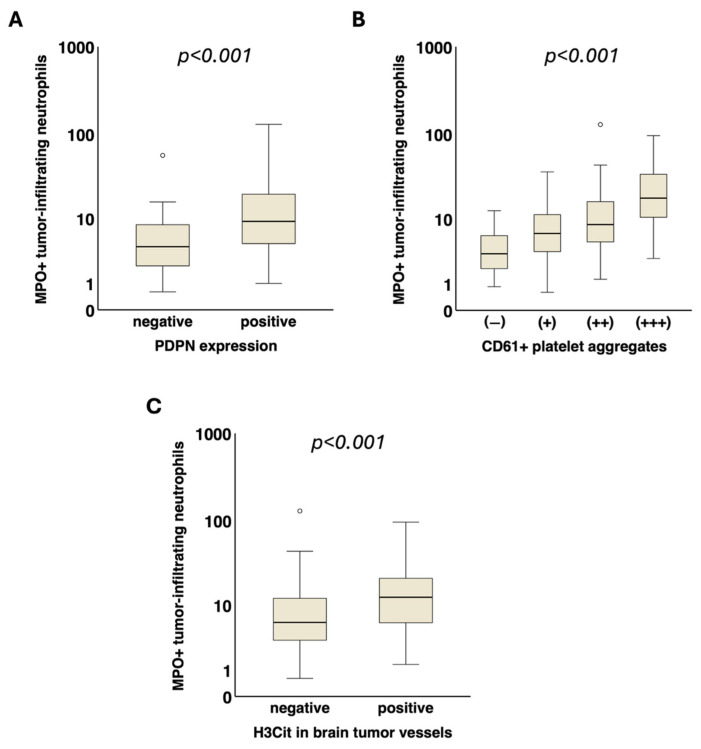
Association of tumor-infiltrating neutrophils with local prothrombotic characteristics in glioma. Tumor-infiltrating MPO+ neutrophils (cells/mm^2^) were significantly associated with increased (**A**) podoplanin expression (*p* < 0.001) and (**B**) intravascular CD61+ platelet clusters (*p* < 0.001) in the tumor microenvironment. (**C**) Furthermore, the density of tumor-infiltrating MPO+ neutrophils was also linked to the presence of H3Cit in tumor vessels (*p* < 0.001). MPO, myeloperoxidase. H3Cit, citrullinated histone H3. PDPN, podoplanin. ° = outlier.

A significant association of tumor-infiltrating MPO+ neutrophils with intratumoral podoplanin expression (PDPN− vs. PDPN +, median [Q1–Q3]: 4.3 [2.2–8.5] vs. 9.2 [4.7–19.9] cells/mm^2^, *p* < 0.001) as well as with intravascular platelet clusters (CD61− vs. CD61+ vs. CD61++ vs. CD61+++, median [Q1–Q3]: 3.4 [1.8–6.2] vs. 6.5 [3.7–11.5] vs. 8.5 [4.7–17.5] vs. 17.9 [8.3–36.3] cells/mm^2^, *p* < 0.001) was detected (Figure 3).

Tumor-infiltrating MPO+ neutrophils (low vs. high, cutoff: median = 7.5 cells/mm^2^) were not significantly associated with following blood parameters: D-dimer (MPO low vs. high, median [Q1–Q3]: 0.61 [0.34–1.01] vs. 0.64 [0.34–2.02] µg/mL, *p* = 0.518), platelets (MPO low vs. high, median [Q1–Q3]: 247 [197–317] vs. 236 [188–314] G/L, *p* = 0.398), soluble P-selectin (MPO low vs. high, median [Q1–Q3]: 37.0 [28.8–50.4] vs. 41.3 [29.3–50.3] ng/mL, *p* = 0.577), neutrophils (MPO low vs. high, median [Q1–Q3]: 5.6 [3.6–9.4] vs. 5.8 [3.7–8.3] G/L, *p* = 0.649) and leukocytes (MPO low vs. high, median [Q1–Q3]: 8.1 [5.7–11.7] vs. 7.6 [5.8–10.6] G/L, *p* = 0.684). The association of several laboratory parameters with different glioma subgroups based on podoplanin and tumor-infiltrating MPO+ neutrophils is listed in Supplementary Table S2.

Patients with H3Cit+ vessels within the brain tumor tissue showed significantly higher levels of tumor-infiltrating MPO+ neutrophils compared to those with H3Cit− vessels (median [Q1–Q3]: 12.5 [5.9–22.0] vs. 6.0 [3.3–12.3] cells/mm^2^, *p* < 0.001) (Figure 3).

### 3.4. Association of NETs in Tumor Vessels and Tumor-Infiltrating Neutrophils with Risk of VTE

During a median follow-up time of 404 days, VTE occurred in 21/154 (13.6%) patients. In detail, 11 (7.1%) patients developed PE, while 10 (6.5%) patients suffered from DVT. In 1-Kaplan–Meier analysis, the cumulative incidence of VTE after 6-month, 12-month, and 24-month was 10.3%, 14.0%, and 16.9%, respectively. No significant differences in age (median [Q1–Q3]: 51 [41.5–67] vs. 55 [44–66] years, *p* = 0.864) or baseline D-dimer levels (0.96 [0.37–3.04] vs. 0.60 [0.34–1.05] µg/mL, *p* = 0.148) were observed in patients with VTE compared to those without VTE. In univariable Cox regression analysis, the hazard ratio (HR) of VTE in glioma patients with increased D-dimer levels was 1.044 (95% confidence interval [CI]: 0.970 to 1.123, *p* = 0.253). In the subgroup analysis of glioblastoma (WHO grade IV), the HR of VTE in patients with elevated D-dimer levels was 1.431 (95% CI: 1.154 to 1.774, *p* = 0.001).

**Figure 4:**
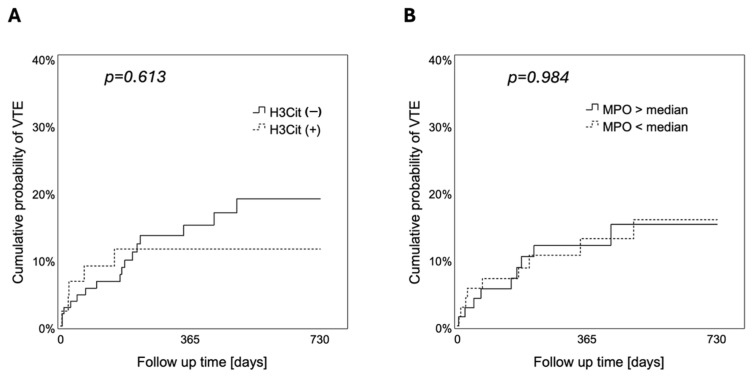
Cumulative probability of VTE according to the presence of NETs in brain tumor vessels and tumor-infiltrating neutrophils. During a 2-year follow-up, neither the presence of (**A**) H3Cit in brain tumor vessels (log-rank, *p* = 0.613) nor (**B**) tumor-infiltrating MPO+ neutrophils (high vs. low, cutoff: median = 7.5 cells/mm^2^) (log-rank, *p* = 0.984) were associated with the risk to develop VTE. VTE, venous thromboembolism. NETs, neutrophil extracellular traps. H3Cit, citrullinated histone H3. MPO, myeloperoxidase.

In univariable Cox regression analysis, the HR of VTE in patients with H3Cit+ brain tumor vessels as compared to those with H3Cit− tumor vessels was 0.772 (95% CI: 0.283 to 2.108, *p* = 0.614) (Supplementary Table S3). In 1-Kaplan–Meier analysis, the cumulative 6-month, 12-month, and 24-month probability of VTE was 11.5%, 11.5%, and 11.5% in patients with H3Cit+ brain tumor vessels compared to 9.8%, 15.0%, and 19.0% in patients with H3Cit− tumor vessels (log-rank, *p* = 0.613) (Figure 4).

In univariable Cox regression analysis, tumor-infiltrating MPO+ neutrophils were not significantly associated with occurrence of VTE (HR [95% CI]: 0.997 [0.969 to 1.027], *p* = 0.862). In 1-Kaplan–Meier analysis, the cumulative 6-month, 12-month, and 24-month probability of VTE was 8.6%, 13.0%, and 15.8% in patients with low density of tumor-infiltrating MPO+ neutrophils compared to 10.3%, 12.0%, and 15.1% in patients with high density of tumor-infiltrating MPO+ neutrophils (low vs. high, cutoff: median = 7.5 cells/mm^2^; log-rank, *p* = 0.984) (Figure 4).

## 4. Discussion

A role for NETs in cancer-related coagulopathy and thrombosis has been proposed in previous studies [36,61]. Here, we were able to show a link between NETs in brain tumor vessels and a local procoagulant phenotype in glioma. Particularly, the NETs-specific biomarker H3Cit was associated with podoplanin expression and intravascular platelet clusters in the tumor microenvironment, indicating a local procoagulant state. Furthermore, systemic procoagulant parameters such as elevated D-dimer blood levels were increased in patients with H3Cit detection in brain tumor vessels. However, we did not find a direct link between VTE risk and the presence of H3Cit. Interestingly, H3Cit in tumor vessels was also associated with tumor-infiltrating neutrophils. Both H3Cit and tumor-infiltrating neutrophils were previously linked to tumor progression in several cancer entities [25,26,40,62,63,64,65]. Whether tumor-infiltrating neutrophils in the brain tumor microenvironment are the predominant source for H3Cit deposits in brain tumor vessels needs to be elucidated in future experimental studies.

NETosis can be induced via different mechanisms, including activated platelets, and vice versa, NETs can trigger platelet activation and interact with the coagulation system [29,41,52,54,66,67,68,69]. In vivo studies revealed that NETs pile up early in growing thrombi [50,66]. The existence of NET components has also been reported in human thrombi [70,71]. In cancer patients, NETs were found in arterial and venous (micro-)thrombi [72,73]. Also in our study, the presence of NETs in brain tumor vessels was linked to intravascular platelet clusters, indicating a prothrombotic phenotype in the brain tumor microenvironment. However, H3Cit detection in brain tumor vessels was not associated with systemic platelet counts. Also, we did not detect an association between H3Cit in tumor vessels and plasma levels of soluble P-selectin, which is a platelet activation marker that is associated with an increased risk of developing VTE in patients with high-grade glioma [12].

Podoplanin is a surface glycoprotein with the ability of platelet activation and patients with podoplanin-expressing brain tumors were linked to an increased risk of VTE development [7,14]. Tumor-expressed podoplanin seems to play a causal role in the formation of platelet aggregates as podoplanin is also associated with intravascular platelet clusters and decreased systemic platelet counts, which is thought to be caused by platelet consumption [7]. Accordingly, mice bearing podoplanin-expressing glioma xenografts also revealed decreased platelet counts in vivo [74]. Here, we demonstrated that podoplanin expression in the tumor tissue is also associated with the presence of NETs in brain tumor vessels supporting the assumption that podoplanin-induced platelet activation could lead to increased NET formation in the tumor microenvironment, which potentially contributes to a hypercoagulable state in patients with brain tumors. In line with this hypothesis, subgroup analysis of our glioma cohort revealed that patients with both podoplanin expression and NETs in tumor vessels had the highest D-Dimer levels. Although we believe that podoplanin upregulation in glioma cells might be the main driver for elevated D-dimer levels and VTE development, we assume that NETs could additionally contribute to a hypercoagulable state in patients with glioma. As expected, lower platelet counts were only observed in podoplanin-positive subgroups, and this was regardless of NETs presence in tumor vessels.

As already mentioned, the exposure of P-selectin on activated platelets and its interaction with PSGL-1 on neutrophils can lead to NETosis [29,53,54]. Another potential mechanism on how podoplanin-induced platelet activation might be involved in NET formation is the release of different mediators upon platelet degranulation. It is well established that platelet activation triggers platelet degranulation which subsequently leads to the release of several cytokines and chemokines [75,76]. These signaling molecules could amplify NETosis and further attract neutrophils [77]. Also, adhesion molecules on activated platelets could enhance the recruitment of neutrophils [78,79]. Supportive to that, intravascular platelet clusters as well as podoplanin expression in our glioma cohort was linked to the density of tumor-infiltrating neutrophils which are a likely source for extracellular traps in the tumor microenvironment. Both podoplanin and tumor-infiltrating neutrophils were previously linked to cancer progression in multiple studies [25,26,80,81,82]. Consistent to our findings, Wang et al. also revealed a correlation between podoplanin and tumor-associated neutrophils in glioma by performing bioinformatic correlation analyses of available databases. Furthermore, podoplanin knockdown in human glioma cell lines resulted in an impaired ability of glioma cells to induce neutrophil infiltration [83]. The exact underlying regulatory mechanisms were not elucidated, although changes in cytokine levels were observed in the podoplanin knockdown cells which might be responsible for the decreased infiltration of neutrophils. The study implied a potential direct interplay between podoplanin and leukocytes, which would be separate from platelets. Whether (tumor-expressed) podoplanin is able to induce NET formation by neutrophils directly or indirectly (via platelet activation) needs to be explored in dedicated studies. Intriguingly, the podoplanin-specific receptor CLEC-2 was not only found on platelets but also on murine neutrophils [84,85]. Nonetheless, recent studies did not detect CLEC-2 on normal human white blood cells [85,86].

Leukocytosis is a well-known risk factor for cancer-associated VTE [87,88]. Additionally, a role for NETs in cancer-related coagulopathy and thrombosis was suggested as well [36,61]. In particular, a recent study of our group revealed an association of systemic H3Cit plasma levels with increased neutrophil blood counts and VTE in a large cohort of cancer patients [89]. Subgroup analysis for brain tumors showed no association between systemic H3Cit levels and risk of VTE. Nevertheless, the study design was not powered for statistical subgroup analysis in distinct tumor entities. Also in the current study, neither H3Cit in brain tumor vessels nor tumor-infiltrating neutrophils were associated with the risk of developing VTE. Depending on the tumor entity, multifactorial pathomechanisms might cause hypercoagulability and thrombosis in cancer patients [90]. In brain tumors, podoplanin (via its ability to activate platelets) seems to play a mechanistic role in VTE development [7]. In addition to intravascular platelet clusters, we revealed that podoplanin is also linked to procoagulant NETs in tumor vessels, representing a local thrombogenic phenotype in glioma that might contribute to a systemic hypercoagulable state.

Inflammation is a hallmark of cancer and plays a key role in tumor progression [91]. Particularly, NET components are able to shield and immobilize circulating tumor cells and thereby enhance cancer cell proliferation at distant sites of the body [33]. Also, procoagulant properties of the tumor promote cancer growth and metastasis, and cancer cells themselves are able to trigger the release of NETs in neutrophils [30,37,38]. Furthermore, the infiltration of the normal brain tissue by glioma cells might be enhanced through local elastase secretion by neutrophils [92]. Interestingly, systemic NETs levels are elevated in cancer patients compared to healthy controls [40,93]. In glioma, systemic NETs levels were also linked to a higher tumor grade [94]. Moreover, NETs and neutrophils in the tumor tissue were linked to worse survival and/or tumor progression in several entities (including glioma) [26,62,63,82,95,96,97,98]. In this study, we further validated a link between tumor-infiltrating neutrophils and an IDH1 wildtype status, which was previously demonstrated by Amankulor et al. in a murine glioma model [99]. Intriguingly, several studies reported a significant difference in immune cell infiltration between IDH wildtype and IDH mutant glioma subgroups [99,100]. Also, the oncogenic product of mutant IDH, namely 2-hydroxyglutarate (2-HG), was able to influence the function of immune cells such as decreasing the activity of T cells [101]. Furthermore, IDH mutation in glioma was associated with decreased leukocyte chemotaxis and the downregulation of several cytokines, which might explain the negative effects of IDH mutation on tumor-infiltrating neutrophils [99,102]. Moreover, in IDH wildtype glioma, it was suggested that TERT mutations might contribute to an increased chemokine expression and subsequent neutrophil enrichment [103]. Nonetheless, we found no association of H3Cit in brain tumor vessels with IDH1 mutation status. Of note, IDH1 mutation is usually present in lower grade glioma and linked to a decreased VTE risk and better prognosis [8,104,105,106].

Several studies reported that impairment of NETosis can inhibit thrombosis and cancer progression: for instance, deficiency of the enzyme PADI4 led to impaired histone citrullination and thereby decreased thrombus formation after inferior vena cava stenosis in mice [107]. Also, PADI4 inhibitors were able to prevent NET formation in mice and humans [108]. DNases are enzymes with the ability to degrade NETs and administration of DNase1 in mice was able to suppress NET formation and venous thrombus development [41,50,109]. As already mentioned, P-selectin on platelets is able to bind to PSGL-1 on neutrophils and signaling through P-selectin/PSGL-1 leads to NETosis and histone citrullination [29,54]. Accordingly, P-selectin and PSGL-1 inhibitors were able to interrupt the interaction of neutrophils with platelets and to decrease NET formation by neutrophils in vivo [29]. Taken together, these experimental studies propose NET-targeting drugs as novel innovative anti-thrombotic and anti-cancer therapies. However, future clinical studies are necessary in order to evaluate the efficacy and safety of these options, particularly regarding the impairment of immunological functions and bleeding risk.

Several limitations of the current study need to be discussed. Our results demonstrate a strong association between NETs in brain tumor vessels and a local prothrombotic phenotype, based on podoplanin expression and intravascular platelet clusters in the tumor microenvironment. Based on these findings, we propose that tumor-expressed podoplanin might be a player in triggering the release of NETs through activated platelets. However, we do not provide experimental data in order to validate our observation. Nonetheless, we provide a large cohort of brain tumor patients, including clinical data and prospective VTE events. Even though H3Cit in brain tumor vessels was linked to a prothrombotic phenotype in glioma, no direct association of H3Cit with VTE occurrence was found. Previously, it was reported that NET levels might be dynamic as NETs are predominantly detected at early stages of thrombus formation and therefore might degrade over time, which could be a confounder in detecting a potentially significant link between VTE events and H3Cit in our patient cohort [71,109,110]. So far, due to a lack of standardized protocols, it remains difficult to detect and quantify NETs directly [61]. Previous studies established H3Cit as a specific marker for NET formation, showing more accuracy as compared to other NET biomarkers that could originate from other sources [111,112]. For instance, cell-free DNA (cfDNA) can also originate from other apoptotic cells (including cancer cells). Furthermore, NE and MPO reflect predominantly neutrophil activation, which does not necessarily result in the release of NET components [110,113,114,115]. Thus, H3Cit was chosen to reflect NET formation within brain tumors in the current study. Yet, it has to be acknowledged that H3Cit and extracellular traps can also originate from other cell types, such as eosinophils or monocytes [116]. In the current study, we used the antibody ab5103 from Abcam, which is a common antibody used in multiple other investigations, in order to detect H3Cit via immunostaining methods [97,98]. To identify tumor-infiltrating neutrophils, we performed immunohistochemical staining with an antibody against MPO. Of note, MPO is an enzyme that is predominantly present in neutrophils but can also be present in small amounts in monocyte lysosomes [117,118,119]. However, we used the Definiens Software (Definiens tissue Studio V.4.4.3, Definiens AG, Munich, Germany) for automatic quantification of MPO+ neutrophils according to specific morphological criteria. Also, we used the same MPO antibody for neutrophil immunostaining that was previously established by Perez-de-Puig et al. in order to identify neutrophils in the brain of stroke patients [120]. Moreover, it needs to be mentioned that we only used an anti-CD61 antibody in order to detect platelets in tumor vessels, which is able to stain all platelets and does not differentiate between activated or resting platelets. However, we observed that predominantly platelet clusters in thrombotic tumor vessels stained highly positive for CD61, probably due to the increased density of platelets within platelet aggregates.

Finally, it also needs to be acknowledged that the diagnosis and grading of gliomas in this study were conducted according to the 2007 WHO classification of CNS tumors, as patients were recruited before the last updated WHO classifications [57,58,59]. The IDH1 R132H mutation status of our patient cohort was determined retrospectively. Overall, the majority of gliomas were classified as WHO grade IV and had an IDH1 wildtype status.

## 5. Conclusions

In conclusion, we revealed that the presence of NETs in tumor vessels was strongly linked to a local prothrombotic phenotype in glioma. However, NETs were not associated with VTE occurrence. Taken together, our findings support the assumption that tumor-expressed podoplanin (via its ability to activate platelets) might be potentially involved in triggering NET release by neutrophils, leading to a synergistic thrombogenic effect. However, future experimental studies are needed to elucidate the underlying mechanisms of our clinical observation. Additionally, novel drugs targeting either NETs or podoplanin could represent innovative therapeutic options for treating thrombosis in (brain) cancer patients.

## Figures and Tables

**Table 1:** Baseline characteristics of the study population (*n* = 154).

Characteristics	
**Median age at study entry (25th–75th percentile)**	55 [44–66]
**Sex, *n* (%)**	
Male	95 (61.7%)
Female	59 (38.3%)
**Newly diagnosed, *n* (%)**	137 (89%)
**IDH1 status, *n* (%)**	
IDH1 mutation	33 (21.4%)
IDH1 wildtype	121 (78.6%)
**Podoplanin expression in glioma, *n* (%)**	
PDPN−	49 (31.8%)
PDPN+	105 (68.2%)
**Intravascular CD61+ platelet clusters, *n* (%)**	
CD61−	27 (17.5%)
CD61+	59 (38.3%)
CD61++	33 (21.4%)
CD61+++	35 (22.7%)

IDH1 = isocitrate dehydrogenase 1, PDPN = podoplanin.

## Data Availability

The datasets generated during and/or analyzed during the current study are available from the corresponding author on reasonable request.

## Supplementary Materials

The following supporting information can be downloaded at: https://www.mdpi.com/article/10.3390/cancers17193141/s1, Table S1: Association of laboratory parameters with glioma subgroups based on H3Cit in tumor vessels and podoplanin expression. Table S2: Association of laboratory parameters with glioma subgroups based on tumor-infiltrating MPO+ neutrophils and podoplanin expression. Table S3: Univariable and multivariable Cox regression analyses of PDPN expression, H3Cit in brain tumor vessels and tumor-infiltrating MPO+ neutrophils with the risk of VTE in patients with glioma. Figure S1: Representative immunohistochemical brain tumor samples of (**A**) IDH1 wildtype, (**B**) IDH1 R132H mutation (**C**,**D**) podoplanin expression and (**E**,**F**) intravascular CD61+ platelet clusters. Figure S2: Representative immunohistochemical samples of (**A**,**B**) H3Cit+ brain tumor vessels and (**C**) tumor-infiltrating MPO+ neutrophils.

## Abbreviations

The following abbreviations are used in this manuscript:

CATS	Vienna Cancer and Thrombosis Study
cfDNA	Cell-free DNA
CLEC-2	C-type lectin-like receptor 2
CNS	Central nervous system
FFPE	Formalin-fixed and paraffin-embedded
H3Cit	Citrullinated histone H3
HR	Hazard ratio
IDH1	Isocitrate dehydrogenase 1
MPO	Myeloperoxidase
MUV	Medical University of Vienna
NE	Neutrophil elastase
NETs	Neutrophil extracellular traps
PADI4	Peptidylarginine deiminase 4
PDPN	Podoplanin
PSGL-1	P-selectin glycoprotein ligand-1
Q	Quartile
VTE	Venous thromboembolism
WHO	World Health Organization
