# Supplementary material for: The Presence of Neutrophil Extracellular Traps (NETs) in Brain Tumor Vessels Is Linked to Platelet Aggregates and Podoplanin in the Tumor Microenvironment
Source: Cancers (Basel). 2025 Sep 27;17(19):3141. doi: 10.3390/cancers17193141 (PMC12523597; doi:10.3390/cancers17193141)
Supplement: Supplementary file 1 [file cancers-17-03141-s001.zip › cancers-3822588-supplementary.pdf]

The presence of neutrophil extracellular traps (NETs) in brain tumor vessels is linked to platelet aggregates and podoplanin in the tumor microenvironment

Supplementary Material

Table S1: Association of laboratory parameters with glioma subgroups based on H3Cit in tumor vessels and podoplanin expression

Laboratory parameter	H3Cit-/PDPN- median [Q1-Q3] 0	H3Cit-/PDPN+ median [Q1-Q3] 1	H3Cit+/PDPN- median [Q1-Q3] 2	H3Cit+/PDPN+ median [Q1-Q3] 3	p-value
D-dimer [µg/ml]	0.37 [0.22-0.61]	0.84 [0.43-1.88]	0.34 [0.21-3.15]	0.94 [0.51-2.77]	**<0.001**
Platelets [G/L]	283 [240-362]	213 [173-265]	335 [318-350]	237 [186-308]	**<0.001**
sP-selectin [ng/ml]	37.5 [28.0-51.0]	36.8 [28.7-47.6]	43.0 [27.7-57.8]	38.0 [30.0-53.0]	0.862
Leukocytes [G/L]	6.7 [5.3-10.5]	8.4 [6.2-11.2]	7.6 [7.0-11.2]	8.4 [6.4-12.1]	0.145
Neutrophils [G/L]	4.4 [3.2-8.5]	6.2 [3.8-9.1]	6.1 [4.4-11.0]	6.0 [4.4-9.7]	0.190

PDPN = podoplanin, sP-selectin= soluble P-selectin, Q=quartile
H3Cit = citrullinated histone H3

Table S2: Association of laboratory parameters with glioma subgroups based on tumor-infiltrating MPO+ neutrophils and podoplanin expression.

Laboratory parameter	MPO-/PDPN- median [Q1-Q3] 0	MPO-/PDPN+ median [Q1-Q3] 1	MPO+/PDPN- median [Q1-Q3] 2	MPO+/PDPN+ median [Q1-Q3] 3	p-value
D-dimer [µg/ml]	0.45 [0.25-0.65]	0.89 [0.46-1.89]	0.32 [0.21-0.63]	0.84 [0.43-2.13]	**<0.001**
Platelets [G/L]	303 [244-355]	212 [175-268]	307 [231-352]	232 [185-284]	**<0.001**
sP-selectin [ng/ml]	37.6 [28.8-52.9]	35.5 [28.5-45.0]	44.5 [26.8-54.1]	41.3 [29.5-49.1]	0.867
Leukocytes [G/L]	6.9 [5.3-10.0]	10.3 [6.3-13.5]	6.3 [5.2-10.9]	7.9 [6.2-10.6]	0.059
Neutrophils [G/L]	4.6 [3.3-8.5]	7.2 [3.6-11.2]	3.7 [3.1-8.0]	6.0 [4.3-8.3]	0.096

PDPN = podoplanin, sP-selectin= soluble P-selectin, Q=quartile
MPO = myeloperoxidase, cut-off median: MPO- = low, MPO+ = high.

Table S3: Univariable and multivariable cox-regression analyses of PDPN expression, H3Cit in brain tumor vessels and tumor-infiltrating MPO+ neutrophils with the risk of VTE in patients with glioma.

	HR	95%CI	p value
**Univariable analyses**			
PDPN expression	3.904	1.141-13.362	0.030
H3Cit+ brain tumor vessles	0.772	0.283-2.108	0.614
MPO+ tumor-infiltrating neutrophils	0.997	0.969-1.027	0.862
**Multivariable analyses**			
**PDPN expression**			
Adjusted for D-dimer	3.671	1.060-12.712	0.040
**H3Cit+ brain tumor vessels**			
Adjusted for age	0.767	0.280-2.099	0.605
Adjusted for D-dimer	1.011	0.367-2.787	0.983
Adjusted for PDPN expression	0.574	0.208-1.587	0.285
Adjusted for IDH1 mutation status	0.676	0.247-1.848	0.445
**MPO+ tumor-infiltrating neutrophils**			
Adjusted for age	0.997	0.969-1.027	0.864
Adjusted for D-dimer	0.998	0.971-1.026	0.894
Adjusted for PDPN expression	0.988	0.955-1.023	0.507
Adjusted for IDH1 mutation status	0.989	0.956-1.022	0.508

HR = hazard ratio, CI = confidence interval, PDPN = podoplanin, H3Cit = citrullinated histone H3, MPO = myeloperoxidase, IDH1 = isocitrate dehydrogenase 1

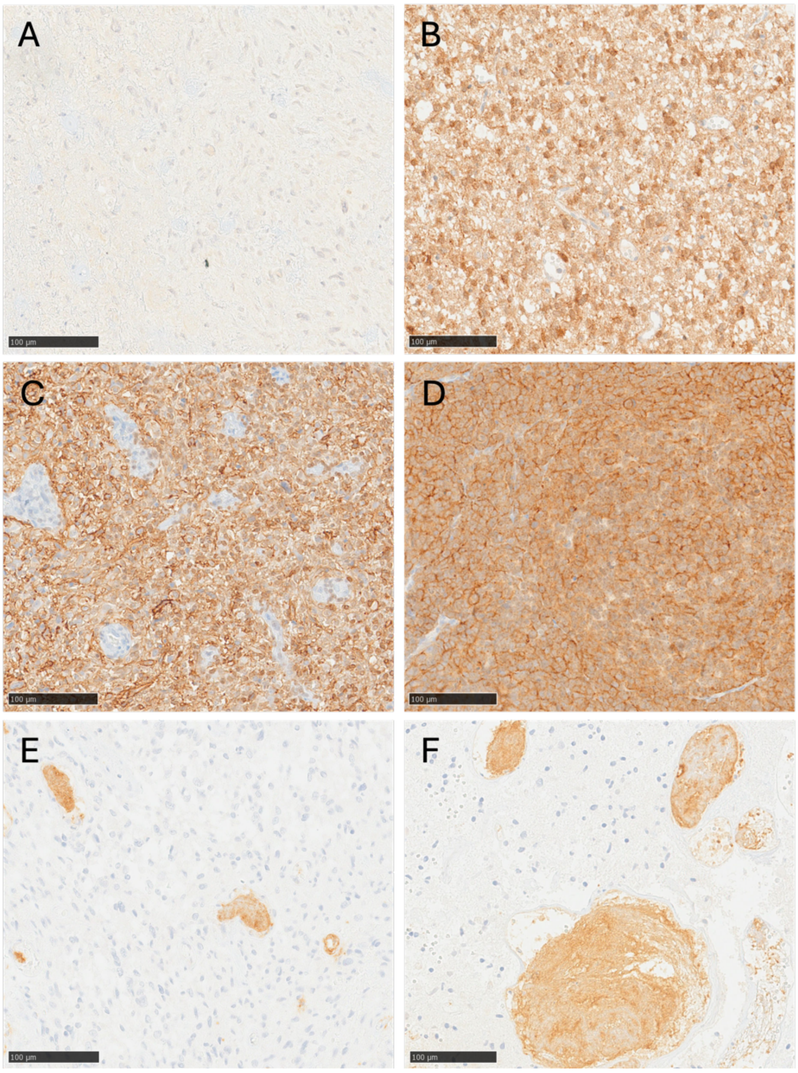

Figure S1: Representative immunohistochemical brain tumor samples of (A) IDH1 wildtype, (B) IDH1 R132H mutation (C,D) podoplanin expression and (E,F) intravascular CD61+ platelet clusters. Full scale bars represent 100 µm.

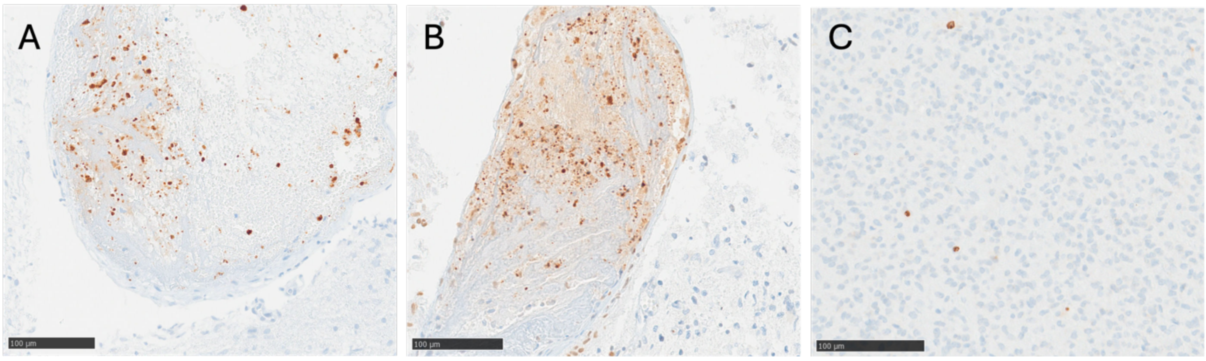

Figure S2: Representative immunohistochemical samples of (A,B) H3Cit+ brain tumor vessels and (C) tumor-infiltrating MPO+ neutrophils. Full scale bars represent 100 µm.
